# Supplementary material for: AKT inhibition impairs PCNA ubiquitylation and triggers synthetic lethality in homologous recombination-deficient cells submitted to replication stress
Source: Oncogene. 2019 Jan 31;38(22):4310–24. doi: 10.1038/s41388-019-0724-7 (PMC6756059; doi:10.1038/s41388-019-0724-7)
Supplement: Supplementary file 1 — Supplementary Figures legends [file 41388_2019_724_MOESM1_ESM.docx]

**Supplementary Figure 1: Single antibody vs dual antibody detection of total and ubi-PCNA.**

**A)** U2OS cells were grown in a 24 MW format. 100K cells were seeded per 24 well. 24 h later cells were UV-irradiated (40 J/m2) and 4 or 8 h post UV cells were processed for WB. Sample lysis was performed with Laemmli-buffer, collected in 1.5 ml tubes and boiled for 15 minutes. The total sample volume for each treatment was loaded in a WB and PCNA and ubi-PCNA detection was performed using a monoclonal antibody against PCNA (clone PC10). **B)** U2OS cells were grown in a 96 MW format. 30K cells were seeded per 96 well. 24 h later samples were UV irradiated (40 J/m2) and 4 h post UV samples were processed for WB. Sample lysis was performed in the same MW plate using benzonase (250U/mL). Loading of WB gels was performed directly from the 96 MW plate without boiling. The total sample volume from each well was loaded and the detection of PCNA and ubi-PCNA was performed by the simultaneous incubation with two monoclonal antibodies from cell signaling (mouse α PCNA, clone PC10, and rabbit α ubi-PCNA clone D5C7P).

**Supplementary Figure 2: Identification and validation of the AKT inhibitors** **GSK1581428A and GSK1389063A. A)** Screening results of the 96 MW plate containing compounds GSK1581428A and GSK1389063A, which were named C11 and G8 respectively, due to their positions in the plate. The quantification was performed using the software of the LICOR Odyssey infrared scanner and the inhibitory capacity of the compounds was calculated by comparing to the normalized values of the 8 non-treated (UV-irradiated) of the plate. **B)** U2OS cells were UV irradiated (15 J/m2) and treated with increasing doses of compound C11, from 0.1 μM to 1 μM. 12 h later, the inhibition of PCNA ubiquitylation and AKT activation were analyzed by western blot.

**Supplementary Figure 3: Tools to validate TLS impairment after AKT inhibition. A)** U2OS cells were either transiently transfected or stably transduced with GFP-H- Pol η using lentiviral vectors. A low magnification image (20 X) using a fluorescence microscope was taken to show the homogeneity and levels of GFP-H- Pol η in each case. The transient expression was evaluated after 48 h and the stable expression was evaluated two weeks after lentiviral transduction and puromycin selection. **B)** DAPI images corresponding to Figure 4A, which were used to segment the nuclei prior to γH2AX quantification.

**Supplementary Figure 4: Induced HR deficiencies in the isogenic cell lines generated to evaluate the impact of impaired PCNA ubiquitylation. A)** Western blot showing the efficiency of BRCA1 knockdown using a lentiviral shRNA approach. The pair of isogenic HCT116^p21-/-^ cells obtained (shSCR and shBRCA1) were used in all the experiments shown in Figures 6 and 7. **B)** HR deficiency triggered by the same shRNA used in A) tested with the DR-GFP method. The right panel shows the normalized quantification of HR efficiency in shSCR vs shBRCA1 cells. **C)** SL induction experiments showing the complete UV dose response curve of the experiment shown in Figure 6F. **D)** Results of the survival experiment using the RAD18 shRNA #4, performed in parallel with shRNA #1 (shown in Figure 7D). Statistical analysis shown in panels C and D was performed using ANOVA (*: p ≤ 0.05; **: p ≤ 0.01; ***: p ≤ 0.001).
