# Supplementary figures and images for: AKT inhibition impairs PCNA ubiquitylation and triggers synthetic lethality in homologous recombination-deficient cells submitted to replication stress
Source: Oncogene. 2019 Jan 31;38(22):4310–24. doi: 10.1038/s41388-019-0724-7 (PMC6756059; doi:10.1038/s41388-019-0724-7)

Supplementary Figure 1

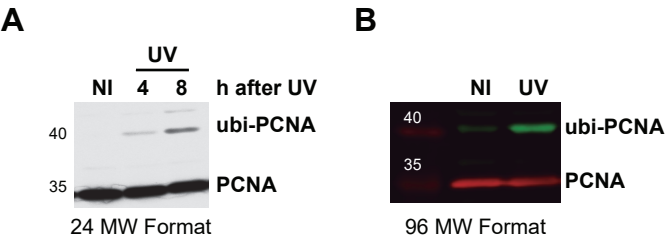

Supplement: Supplementary file 2 — Supplementary Figure 1 [file 41388_2019_724_MOESM2_ESM.pdf]

# Supplementary Figure 2

**A**

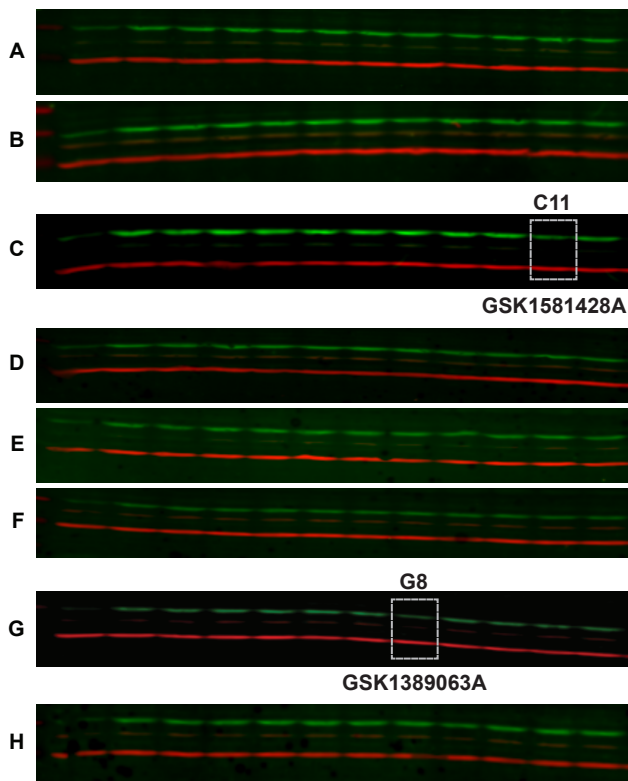

**B**

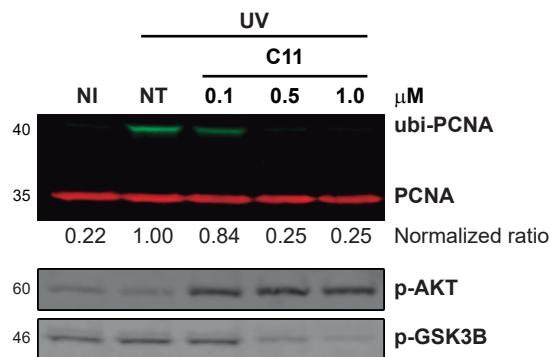

**C**

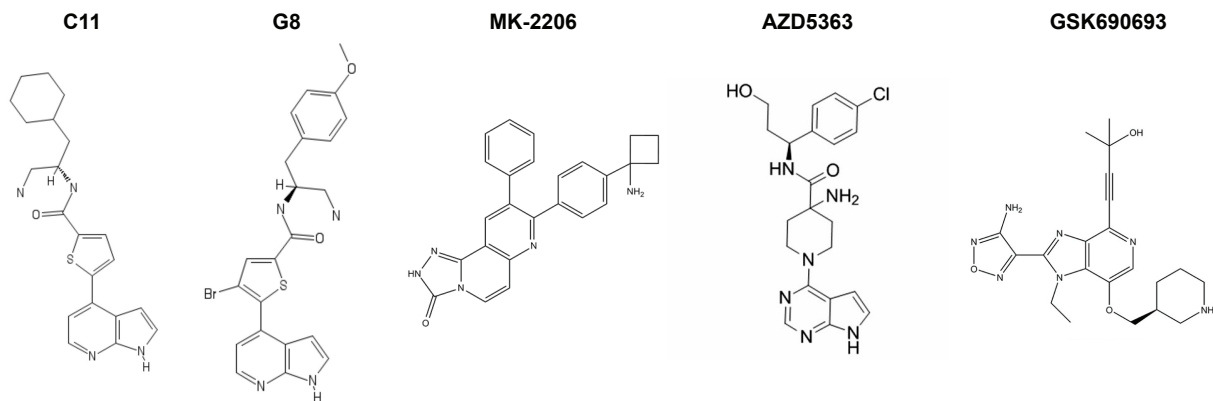

Supplement: Supplementary file 3 — Supplementary Figure 2 [file 41388_2019_724_MOESM3_ESM.pdf]

Supplementary Figure 3

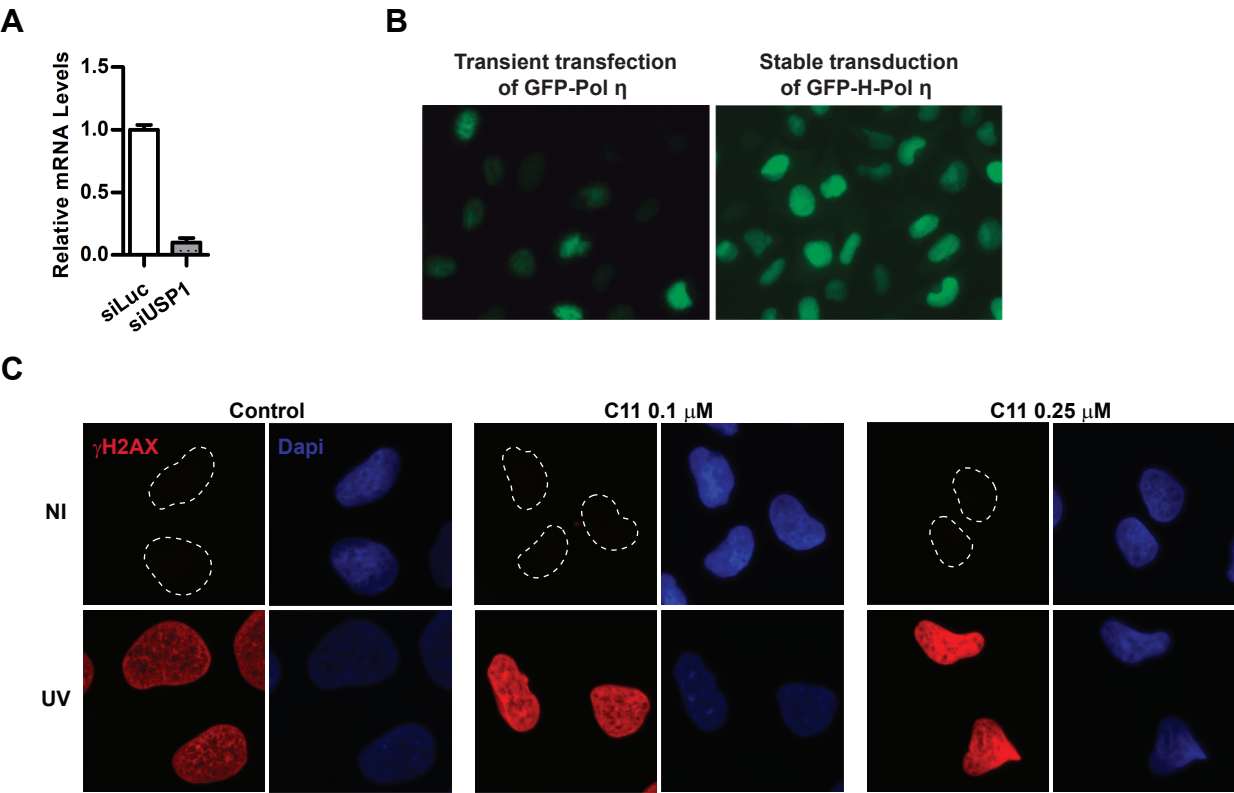

Supplement: Supplementary file 4 — Supplementary Figure 3 [file 41388_2019_724_MOESM4_ESM.pdf]

# Supplementary Figure 4

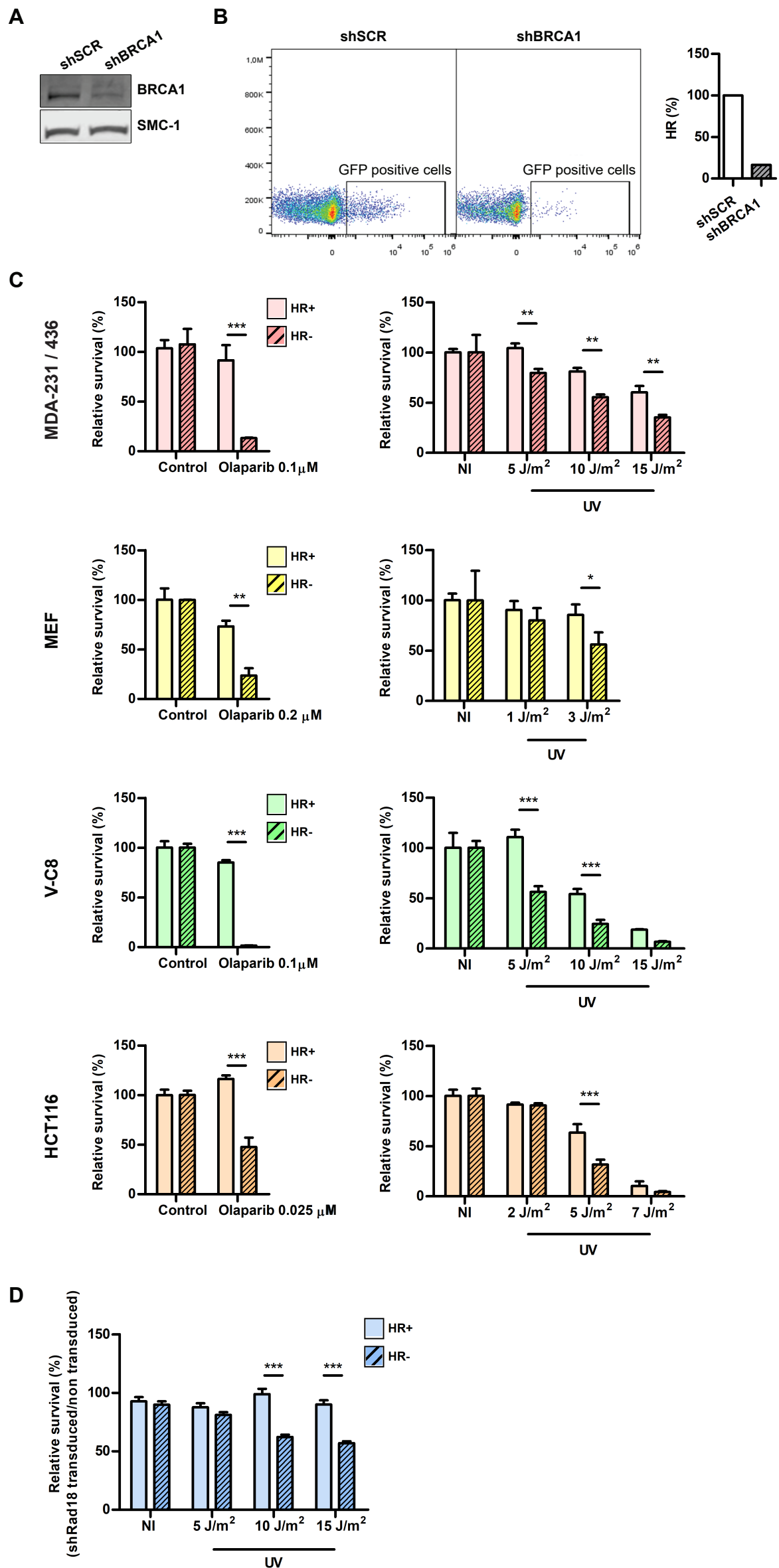

Supplement: Supplementary file 6 — Supplementary Figure 4 [file 41388_2019_724_MOESM6_ESM.pdf]
