# Supplementary material for: AKT inhibition impairs PCNA ubiquitylation and triggers synthetic lethality in homologous recombination-deficient cells submitted to replication stress
Source: Oncogene. 2019 Jan 31;38(22):4310–24. doi: 10.1038/s41388-019-0724-7 (PMC6756059; doi:10.1038/s41388-019-0724-7)
Supplement: Supplementary file 7 — Revised Marked up version of the manuscript [file 41388_2019_724_MOESM7_ESM.pdf]

AKT inhibition impairs PCNA ubiquitylation and triggers synthetic lethality in homologous-recombination-deficient cells submitted to replication stress

Florencia Villafañez[1,2]*, Iris Alejandra García[1,2]*, Sofia Carbajosa[1,2], María Florencia Pansa[1,2], Sabrina Mansilla[3], María Candelaria Llorens[1,2], Virginia Angiolini[1,2], Laura Guantay[1,2], Heinz Jacobs[4], Kevin P Madauss[5], Israel Gloger[6], Vanesa Gottifredi[3], Jose Luis Bocco[1,2] and Gaston Soria[1,2].

***: These authors contributed equally to this work**

1) Centro de Investigaciones en Bioquímica Clínica e Inmunología, CIBICI-CONICET.

2) Departamento de Bioquímica Clínica. Facultad de Ciencias Químicas, Universidad Nacional de Córdoba. Córdoba, Argentina.

3) Fundación Instituto Leloir. Buenos Aires, Argentina.

4) Tumor Biology & Immunology-Netherlands Cancer Institute. Amsterdam, The Netherlands.

5) GlaxoSmithKline - Trust in Science, Global Health R&D Upper Providence, PA, United States.

6) GlaxoSmithKline - Trust in Science, Global Health R&D. Stevenage, United Kingdom.

Correspondence should be addressed to Gaston Soria: gsoria29@gmail.com ; gsoria@fcq.unc.edu.ar

Running title: ubi-PCNA inhibition is synthetic lethal with HR deficiency

Abstract

Translesion DNA Synthesis (TLS) and homologous recombination (HR) cooperate during S-phase to safeguard replication forks integrity. Thus, the inhibition of TLS becomes a promising point of therapeutic intervention in HR-deficient cancers, where TLS impairment might trigger synthetic lethality (SL). The main limitation to test this hypothesis is the current lack of selective pharmacological inhibitors of TLS. Herein, we developed a miniaturized screening assay to identify inhibitors of PCNA ubiquitylation, a key post-translational modification required for efficient TLS activation. After screening a library of 627 kinase inhibitors, we found that targeting the pro-survival kinase AKT leads to strong impairment of PCNA ubiquitylation. Mechanistically, we found that AKT-mediated modulation of PCNA ubiquitylation after UV requires the upstream activity DNA-PKcs, without affecting PCNA-ubiquitylation levels in unperturbed cells. Moreover, we confirmed that persistent AKT inhibition blocks the recruitment of TLS polymerases to sites of DNA damage and impairs DNA replication forks processivity after UV irradiation, leading to increased DNA replication stress and cell death. Remarkably, when we compared the differential survival of HR-proficient vs HR-deficient cells, we found that the combination of UV irradiation and AKT inhibition leads to robust SL-induction in HR-deficient cells. We link this phenotype to AKT ability to inhibit PCNA ubiquitylation, since the targeted knockdown of PCNA E3-ligase (RAD18) and a non-ubiquitylable (PCNA K164R) knock-in model recapitulate the observed SL-induction. Collectively, this work identifies AKT as a novel regulator of PCNA ubiquitylation and provides the proof-of-concept of inhibiting TLS as a therapeutic approach to selectively kill HR-deficient cells submitted to replication stress.

Introduction

The mono-ubiquitylation of PCNA (ubi-PCNA) steeply increases after treatment with DNA damaging agents that induce DNA replication fork stalling, like hydroxyurea, methyl methanesulfonate, cisplatin, aphidicolin and UV irradiation [1–5]. Ubi-PCNA, along with the specialized ubiquitin-binding domains present in the Y-family of polymerases [6], are key players in translesion DNA synthesis (TLS) across damaged DNA templates [7]. Several regulatory factors of ubi-PCNA and TLS have been identified and characterized, like p21, REV1, USP1 and Spartan [8–10]. However, there is a lack of selective pharmacological inhibitors of TLS that could be used to explore the therapeutic potential of TLS inhibition. While some efforts have been made to identify selective inhibitors of TLS polymerases [11], there are no universal TLS inhibitors available. Our previous work with the PCNA-interacting domain of p21 shows that this region blocks PCNA interaction with all the TLS polymerases tested, including Pol eta, Pol iota, Pol kappa and REV1, triggering replication forks stalling and genome instability [12]. Thus, the global and upstream interference of TLS may have a more robust biological effect than the individual targeting of each TLS polymerase. A central hypothesis of this work is that a way to selectively impair TLS, without impacting on critical housekeeping functions of PCNA would be to inhibit PCNA ubiquitylation. Hence, we designed a screening focused on the modulation of PCNA ubiquitylation. We evaluated a library of kinase inhibitors and identified AKT as a regulator of PCNA ubiquitylation.

AKT is an iconic pro-survival kinase that controls essential cellular functions such as growth, proliferation, apoptosis and metabolism [13]. In fact, even before the vast repertoire of AKT targets were identified, multiple groups independently demonstrated the central

involvement of AKT in promoting cell survival [14]. AKT has a well-established anti-apoptotic function, operating both directly, through the phosphorylation of relevant targets such as BAD and caspases, and indirectly, through the transcriptional modulation of pro-survival or pro-apoptotic genes such as IKK/NF-κB and MDM2/p53 [15]. Herein, we describe a new role for AKT in the regulation of PCNA ubiquitylation and TLS. We also show that AKT inhibitors can be used to achieve selective killing of HR-deficient cells in a manner that depends on their ability to inhibit PCNA ubiquitylation.

Results

1- Development of a miniaturized western blot-based screening method to identify PCNA ubiquitylation inhibitors.

The mono ubiquitylated form of PCNA (ubi-PCNA) can be detected by classical western blot using antibodies against total PCNA. However, since the proportion of ubi-PCNA to total-PCNA is low, the detection of ubi-PCNA requires the loading of high protein concentrations, which implies working with samples from 24 MW formats or larger (supp Fig 1A). Moreover, in conditions where the amounts of ubi-PCNA are remarkably lower (i.e. unperturbed or inhibited conditions), the detection of ubi-PCNA requires even larger samples and long exposure times with classical chemiluminescence methods. While such types of experiments are suitable for fundamental research of PCNA biology, they do not provide either the sensitivity range nor the throughput capacity required for screening purposes. In this work, we developed a detection method of ubi-PCNA using two monoclonal PCNA antibodies. We used a novel antibody that detects ubi-PCNA in combination with an antibody that detects total-PCNA (Fig 1A and Supp. Fig 1B). For the detection and quantification of each PCNA form we employ LI-COR technology (Odyssey CLX), which provides a wide sensitivity range for quantification with very low background. This setup allowed us to perform western blots with samples obtained from a single 96 well, making it possible to detect up to a 5-fold induction of ubi-PCNA levels after 12 h of UV irradiation (Fig 1A). The calibration of the method was performed using non-specific PCNA ubiquitylation inhibitors, such as Epoxomicin and MG132 (Fig 1A). These drugs inhibit the proteasome, thus causing accumulation of ubiquitylated proteins and depleting the free

ubiquitin required for normal ubiquitylation reactions [16]. The use of a U2OS stable cell line expressing iRFP and the automatic capture of brightfield images were utilized as quality controls to monitor cell number, intra-well distribution, edge effects and general cytotoxicity (Figure 1B), allowing to screen 80 compounds per 96 MW plate (Figure 1C).

2- Screening with a library of kinase inhibitors

With the goal of identifying novel druggable targets to inhibit PCNA ubiquitylation we performed a screening using a library of 627 ATP-competitive kinase inhibitors provided by GlaxoSmithKline (**PKIS2**: **T**he **P**ublic **K**inase **I**nhibitor **S**et **2**). As shown in Figure 1B, the screening was carried out combining each inhibitor at 1 µM with 15J/m2 of UV irradiation. The cut-off to define a hit was a decrease in the ratio of ubi-PCNA/total-PCNA greater than 3 standard deviations of the average of the 8 UV control samples from each screening plate. 22 hits were identified using this cut-off (Fig 1D). The analysis of databases such as PubChem and ChEMBL, as well as recent publications using the PKIS library allowed us to determine the putative target/s for the top list of hits (Supp table 1).

Among the 22 hits, two related AKT inhibitors (GSK1581428A and GSK1389063A) were identified. For simplicity, we called these compounds C11 and G8 respectively, due to their position on the screening plates (Supp Fig 2A). Early validation experiments confirmed that these compounds were strong inhibitors of PCNA ubiquitylation, leading to ubi-PCNA levels in UV-irradiated cells that were close to the non-irradiated samples (Fig 2A and Supp Fig 2B). The analysis of phospho- and total-AKT confirmed that C11 and G8 were in fact ATP-competitive inhibitors of AKT, promoting both the accumulation of inactive pAKT and the degradation of total AKT (Fig 2B), as it was previously reported for other ATP-competitive

AKT inhibitors [17,18]. In the following experiments, the analysis was focused on C11 due to its remarkable activity and dose response behavior (supp Fig 2B). To rule out potential off-target effects and to assess whether the impairment of PCNA ubiquitylation was indeed a consequence of AKT inhibition, we also evaluated three commercially available AKT inhibitors with different chemical backbones: MK-2206, AZD5363 and GSK690693 (Supp Fig 2C). In all cases, AKT inhibitors significantly impaired PCNA ubiquitylation (Fig 2C). We also confirmed that AKT activity was substantially impaired, since the downstream targets pGSK3b and pPRAS40 abruptly decreased after the treatment with these compounds (Fig 2C). Among these inhibitors, MK-2206 is particularly relevant since it has an allosteric mechanism of action. Such activity was confirmed when analyzing pAKT levels. It was clear that in contrast to the ATP-competitive type of inhibitors that trigger the accumulation of inactive pAKT, MK-2206 abrogated the phosphorylation of AKT, yet leading to similar inhibition of GSK3β and PRAS40 phosphorylation (Fig 2C).

To get genetic evidence to further support the involvement of AKT in the regulation of PCNA ubiquitylation we used siRNAs against AKT. Consistently, targeted AKT silencing recapitulated the effects of pharmacological AKT inhibition, thus confirming AKT participation PCNA ubiquitylation (Fig 2D). Together, the experiments of pharmacological AKT inhibition and siRNA led to the conclusion that AKT promotes PCNA ubiquitylation after UV irradiation.

3- AKT promotes the induction of PCNA ubiquitylation after UV but does not modulate PCNA ubiquitylation in unperturbed cells.

To get further insight into the signaling axis in which AKT promotes the ubiquitylation of PCNA, we explored the inhibition of multiple kinases that could be involved in the direct or indirect activation of AKT in response to UV. Unsurprisingly, the inhibition of the widely characterized upstream kinase PI3K led to a strong inhibition of PCNA ubiquitylation (Fig 3A). This result clearly demonstrates that if pAKT levels are depleted, the induction of ubi-PCNA after UV irradiation is critically impaired. PI3K is involved in AKT activation in response to several physiological ligands[19], but was not directly linked to AKT activation after genotoxic stimuli. Therefore, we explored the DNA damage response Kinases ATM, ATR and DNA PKcs. Interestingly, while both ATM and ATR inhibition did not block the induction of PCNA ubiquitylation after UV, the inhibition of DNA PKcs impaired PCNA ubiquitylation (Fig 3A). DNA PKcs inhibition also showed an additive effect when combined with a suboptimal dose of the AKT inhibitor C11 (Fig 3B), thus suggesting that both kinases are part of the same cellular response to promote PCNA ubiquitylation. Given that AKT is a direct phosphorylation target of DNA PKcs in response to UV irradiation [20], this set of findings indicate that UV-induced DNA damage might be the trigger that activates AKT to promote PCNA ubiquitylation. In line with this notion, we observed that none of the inhibitors that blocked the induction of PCNA ubiquitylation after UV (including the potent AKT inhibitor C11) were able to alter the basal levels of PCNA ubiquitylation of unperturbed cells (Fig 3C). This conclusion was further supported by experiments using siRNAs against the PCNA de-ubiquitinase USP1[21]. Under unperturbed conditions, knockdown of USP1 led to a substantial

increase of PCNA ubiquitylation, which was only slightly attenuated by AKT inhibition (Fig 3D). Therefore, we concluded that AKT is required for efficient PCNA ubiquitylation in response to the replication stress induced by UV irradiation.

4- AKT inhibition impairs the recruitment of the Translesion DNA Synthesis polymerase η to damaged DNA sites.

The next important step was to confirm if the blockage of PCNA ubiquitylation triggered by AKT inhibition suffices to alter functional parameters of Translesion DNA Synthesis (TLS). Since ubi-PCNA is required for efficient targeting of TLS polymerases to sites of DNA damage [22], we initially studied the recruitment of the TLS polymerase η to Damaged DNA sites. To analyse such recruitment in large cell populations, we first cloned a hydrophilic linker (H) between Pol η and GFP, which allowed the expression of high levels of exogenous Pol η without apparent toxicity. Then, we generated a cell line that stably expresses GFP-H-Pol η using lentiviral transduction and puromycin selection (supp Fig 3A). With this cell line, two complementary approaches were used to study the recruitment of Pol η to damaged sites: 1) local UV irradiation through polycarbonate shields and 2) total UV irradiation followed by Triton extraction. The use of polycarbonate shields with 5 µm pores allows the irradiation of discrete areas within the nuclei (Fig 4A), which can be identified by immunofluorescence with antibodies against one of the main types of UV-induced DNA lesions: the cyclobutane pyrimidine dimers (CPDs). Then, within those damaged areas we studied the efficiency of recruitment of GFP-H-Pol η with or without AKT inhibition. While in control conditions GFP-H-Pol η recruitment was observed in essentially every CPD-positive cell, less than 50% of CPD-positive cells showed detectable GFP-H-Pol η recruitment when AKT was inhibited (Fig

4B). To confirm this result, we used a method based on total UV irradiation (Fig 4C). In this case the complete MW plate was irradiated with a lower UV dose and, prior to fixation, the cells were treated with a short pulse of PBS containing 0.1% Triton. As such, the chromatin bound fraction of GFP-H-Pol η remains loaded at DNA damage areas while the soluble fraction is washed away. To quantify the total GFP-H-Pol η fluorescence from the cells, we developed an Image J Macro that uses DAPI for nuclei identification and segmentation. After using this Macro to quantify several images of each condition, we concluded that AKT inhibition severely impairs GFP-H-Pol η chromatin retention after UV irradiation (Fig 4D). Thus, AKT inhibition impairs two functional parameters of TLS: PCNA ubiquitylation and TLS polymerases recruitment to DNA damage sites.

The data available so far indicated that TLS is impaired when AKT is inhibited after UV irradiation. To obtain further evidence to consolidate this conclusion, we performed DNA combing experiments to study DNA replication fork processivity after UV. If TLS activation is affected after AKT inhibition, DNA replication fork processivity should be impaired. Sequential pulses of CldU and IdU were performed in UV-irradiated cells (Fig 4E). The measurement of the track length of dual color DNA fibers allowed us to determine the relative processivity of DNA replication forks. We observed a significant decrease in the average speed of ongoing DNA elongation when UV irradiated cells were treated with AKT inhibitors (Fig 4F). Such impaired processivity of replication forks is in line with the effect of AKT inhibition on ubi-PCNA levels and on the recruitment of the TLS polymerase η to damaged sites (Figs 2 and 4). Together, these results indicate that TLS activity after UV

depends on AKT function, and therefore, that AKT inhibition could be used to inhibit TLS activation.

5- AKT inhibition induces replication-associated DNA damage and cell death after UV.

Since AKT promotes TLS activation, we figured that AKT inhibition in UV-irradiated cells should lead to increased replication stress, DNA damage and potentially cell death. To test this hypothesis, we first explored the phosphorylation of histone H2AX as a broad marker of DNA damage. Interestingly, while γH2AX did not increased after AKT inhibition in non-irradiated cells, it significantly increased after UV irradiation (Fig 5A and supp Fig 3B), thus suggesting that the increased H2AX phosphorylation in these cells could be linked to the inhibition of TLS. Cell cycle analysis revealed that AKT inhibition did not induced a substantial change in the profile of non-irradiated cells (Fig 5B), triggering only a small increase in the sub-G1 population (Fig 5B). As expected, UV irradiation led to a noticeable accumulation of cells in S-phase as a result of replication stress (Fig 5B). Remarkably, when UV irradiation was combined with AKT inhibition, a substantial increase in the sub-G1 population was observed (Fig 5B), thus suggesting the rapid activation of the apoptotic program in these cells. The confirmation of cell death induction by UV irradiation combined with AKT inhibition was performed using the cell death stain sytox red, which showed a pronounced decrease of cell viability (Fig 5C). Taken together, these experiments confirmed that AKT inhibition is more toxic in the context of UV irradiation, presumably due to its impact on PCNA ubiquitylation and TLS activation.

6- AKT inhibition after UV triggers synthetic lethality in homologous recombination deficient cells

The main goal of this work was to perform *proof-of-concept* experiments to test the hypothesis that inhibiting PCNA ubiquitylation should become increasingly toxic in cellular backgrounds with deficient homologous recombination repair (HR). To test this, we used a method developed in our lab, in which HR-proficient and HR-deficient cells are co-cultured in the same well, followed by the quantification of the percentage of cells of each population that survive the treatment (Fig 6A). In this experimental setting, HR-deficiency in isogenic genetic backgrounds is artificially induced by lentiviral transduction of shRNAs against BRCA1. The downregulation of BRCA1 was assessed by western blot and the inhibition of HR was confirmed using the direct repeats method from Maria Jasin´s Lab (Supp Fig 4A and B). Since HR-proficient (shSCR) and HR-deficient cells (shBRCA1) are tagged with different fluorescent proteins, the relative viability of both cell populations is determined at the end of the experiment by cell counting using automated flow cytometry (Fig 6A). This assay allows to discriminate if a given treatment (or a combinations of treatments) is equally or selectively toxic for a given genetic background (Fig 6A). As a positive control of synthetic lethality induction we used Olaparib (Fig 6B), a well characterized PARP inhibitor that induce SL in HR-deficient cells [23]. Remarkably, when combining UV irradiation and AKT inhibition, a strong induction of SL was observed in a UV-dose dependent manner using C11 and other AKT inhibitors (Fig 6C). To exclude potential artifacts derived from the co-culture of BRCA1-proficient and deficient populations we also performed a clonogenic assay using single cell cultures. We observed a decreased

clonogenic potential in C11-treated HR-deficient cells when compared to C11-treated HR proficient cells (Fig 6D).

To validate this SL phenotype in different genetic backgrounds, we performed survival experiments with multiple pairs of isogenic and non-isogenic cell lines (HR-proficient vs HR-deficient). We used a pair of triple negative breast cancer cell lines (MDA-MB 231-BRCA1wt vs MDA-MB 436-BRCA1KO), a set of wt MEF (shSCR vs shBRCA1), a hamster BRCA2 KO cell line with its reconstituted counterpart (VC8 vs VC#13) and a pair HCT116 cells (shSCR vs shBRCA2). A *sine qua non* to use these cell lines was the selective sensitivity to Olaparib in the BRCA-deficient counterpart of each pair (supp Fig 4C). In all cell lines, AKT inhibition impaired PCNA ubiquitylation induction (Fig 6E) and triggered SL in the HR-deficient counterpart of each cell pair after UV (Optimal SL doses are depicted in Fig 6F and full dose-response panels in supp Fig 4C).

Taken together, these results allowed us to conclude that AKT inhibition in the context of UV irradiation triggers synthetic lethality in HR-deficient cells.

7- Direct inhibition of PCNA ubiquitylation is synthetic lethal in HR-deficient cells submitted to replication stress

While our data clearly showed that AKT inhibition impairs PCNA ubiquitylation (Fig 2) and triggers SL in HR-deficient backgrounds after UV (Fig 6), a direct causality between ubi-PCNA decrease and SL induction cannot to be claimed given the multiple roles of AKT in cell survival pathways[13]. Hence, we used additional experimental models to study the contribution of PCNA ubiquitylation to the cell survival of UV-irradiated HR-deficient cells. Our initial approach was to downregulate RAD18, the E3-ligase in charge of PCNA mono-

ubiquitylation[24]. We used a lentiviral shRNA transduction protocol to knockdown RAD18. We tested 4 different shRNA sequences and selected two that promoted strong RAD18 downregulation and that severely impaired PCNA ubiquitylation after UV (Fig 7A and B). Then, we adapted our SL induction assay to assess the impact of RAD18 knockdown in the differential survival linked to UV irradiation and HR proficiency (Figure 7C). Increased sensitivity to UV in shRAD18-transduced cells was observed exclusively in the HR-deficient cell line, but not in the isogenic HR-proficient cell line (Fig 7D and supp Fig 4D). While this result indicates that the inhibition of PCNA ubiquitylation could be triggering SL in HR-deficient cells exposed to UV, the fact that RAD18 might be ubiquitylating other HR relevant targets complicates the drawing of a simple cause-effect conclusion. Therefore, we also used a Knock-in MEF model, where wt PCNA was replaced by a non-ubiquitylable version harboring the point mutation K164R[25]. We first confirmed that $PCNA^{K164R}$ cells do not display induction of PCNA ubiquitylation after UV (Fig 7E). Later on, we performed survival experiments in which BRCA1 was downregulated by lentiviral shRNAs in both $PCNA^{WT}$ and $PCNA^{K164R}$ MEFs. Remarkably, a strong SL induction was observed in BRCA1-deficient $PCNA^{K164R}$ cells (Fig 7F). These results consolidate our findings that ubi-PCNA impairment leads to SL induction in HR-deficient cells, and demonstrates that a targeted blockage of PCNA ubiquitylation by different experimental approaches suffices to induce SL. While these data put forward a novel therapeutic strategy to selectively kill HR-deficient cells, an obvious limitation from these experiments is that UV irradiation cannot be used as a sensitizer in a clinical setup. Thus, we decided to evaluate cisplatin, a well characterized replication stress inducer that requires active TLS for DNA damage processing [26]. Strikingly,

the HR-deficient counterpart of both shRAD18 transduced cells and $PCNA^{K164R}$ MEF displayed increased sensitivity to cisplatin (Fig 7G and H, respectively). Collectively, these results unveil the potential of inhibiting PCNA ubiquitylation as a therapeutic strategy to sensitize HR-deficient cells to treatments that induce replication stress and activation of TLS.

Discussion

A new function for AKT in the promotion of cell survival: the regulation of DNA damage tolerance.

A central regulatory affair for cell survival is to balance DNA repair and apoptosis induction in response to DNA damage load. Since DNA damage is an unceasing threat arising from both endogenous and exogenous sources [27], DNA repair pathways are unable to cope with every single DNA damage event in real time, in particular at sensitive points of the cell cycle such as S-phase [28]. Hence, a series of specialized mechanisms have evolved to deal with unrepaired DNA damage in S-phase to promote cell survival and to increase the time-frame for DNA repair mechanisms to attend the damage, which are collectively referred as DNA damage tolerance pathways [22,29]. The most well characterized tolerance pathways are template switch and TLS, two related mechanisms to deal with damaged DNA in S-phase which share a common player: ubiquitylated PCNA [30]. While TLS involves mono ubi-PCNA and template switch involves poly ubi-PCNA, the poly-ubiquitylation of PCNA requires the initial mono-ubiquitylation at the same K164 residue [30]. Thus, the inhibition of PCNA mono-ubiquitylation also implies the inhibition of PCNA poly-ubiquitylation, and the potential impairment of both DNA damage tolerance pathways.

The findings of this paper unveil a novel pro-survival role for AKT, the modulation of PCNA ubiquitylation. Our data not only consistently demonstrate such new role by pharmacological and siRNA-mediated inhibition of AKT (Fig 2 and Supp Fig 2), but also prove that blocking PCNA ubiquitylation through AKT inhibition modifies functional parameters of TLS, such as TLS polymerases recruitment to damage sites, replication forks processivity,

replication stress induction and cell survival after UV irradiation (Figs 4 and 5). Regarding the upstream players of AKT activation in the context of replication stress, there is compelling evidence suggesting that the master DNA Damage Response (DDR) kinase DNA-PKcs regulates AKT through direct phosphorylation at Ser 473 in response to ionizing DNA damage [31–33] and UV irradiation [20]. Our results indicate that DNA PKcs is the DDR kinase that might be coordinating AKT activation to promote PCNA ubiquitylation in response to UV. Given the results with the inhibitor LY294002 (Fig 3A), we cannot exclude that PI3K also participates in the same or a parallel pathway that DNA PKcs in response to replication stress. Nonetheless, a clear conclusion from our results is that the basal levels of PCNA ubiquitylation are not affected by any of these kinases, including AKT itself (Fig 3C). Thus, it is feasible that a replication stress-triggered axis involving DNA PKcs and AKT is activated to promote PCNA ubiquitylation and TLS when damaged DNA accumulate in cells.

An important question that remains open for future studies is the identity of the downstream AKT targets responsible of promoting PCNA ubiquitylation, and whether this occurs directly through the phosphorylation of relevant substrates by AKT or if it requires a more complex signaling cascade. Potential starting points for this research are some of the kinases identified as hits in the screening performed herein, such as IKK and p38 (supplementary table 1). Nonetheless, omics approaches such as RNAseq or phosphor-proteomics comparing UV-irradiated +/- AKT inhibitors will most likely be required to tackle this issue in a more comprehensive manner. It will also be important to study in more detail the TLS regulatory proteins RAD18 and REV1 since two lines of evidence suggest that an axis involving AKT/REV1/RAD18 could modulate PCNA ubiquitylation. First, a recent report

showed that increased REV1 levels boost PCNA ubiquitylation after UV irradiation through the direct interaction with RAD18 [34]. Second, the deletion mutant of yeast AKT homolog Sch9 displays reduced levels of REV1 and impaired TLS [35].

In this paper we establish for the first time a connection between AKT and DNA damage tolerance through TLS activation. Interestingly, AKT has also been linked to the modulation of DNA repair pathways to promote cell survival yet compromising genome stability, which can be considered as additional DNA damage tolerance strategies. One clear example is the modulation of mismatch repair (MMR) by AKT, through the control of the stability and localization of the MMR protein hPMS2 [36]. Since the induction of apoptosis by base adducts like O6MeG requires active MMR, the attenuation of MMR by AKT might promote cell survival in this context, yet increasing the chances of acquiring mutations [37]. Another example of the activation of error-prone DNA repair mediated by AKT to promote cell survival is the stimulation of non-homologous end joining, NHEJ, by collaborating with DNA PKcs (reviewed in [37]). Hence, our work builds up on an emerging role for AKT and DNA PKcs in DNA damage tolerance, which will be of great importance to understand the mechanisms that govern the choice between cell survival and cell death triggered in response to DNA damage.

TLS inhibition as a novel therapeutic strategy against HR-deficient cancers.

Reports by many different groups showed that PCNA ubiquitylation and ubiquitin binding domains on TLS polymerases are less critical for cell survival in mammalian cells than in yeast. On the one hand, it was reported by different groups that to boost the sensitivity

associated with TLS inhibition in different cellular models, a concomitant inhibition of checkpoint activation by caffeine treatment is required [38,39], thus showing that the intricated DDR network in mammals is able to buffer TLS impairment. On the other hand, critical evidence of the relevance of PCNA ubiquitylation came with the generation of a mammalian knock-in model of non-ubiquitylable PCNA [25,40]. The PCNA K164R MEFs obtained showed increased -yet moderate- UV sensitivity when compared to wt MEFs, in particular at low UV doses [40]. Such mild UV-triggered sensitivity observed in mammalian cells in comparison to the extreme phenotype observed in yeast [1], along with the central housekeeping roles of PCNA in DNA replication [41,42], discouraged the field from further exploring the therapeutic potential of targeting PCNA ubiquitylation. However, the possibility that some genetic backgrounds could depict enhanced sensitivity to TLS inhibition remained almost completely unexplored. In such context, the driving hypothesis of this work was that the UV sensitivity associated to PCNA ubiquitylation inhibition could become much stronger in DNA repair-deficient contexts. In particular, we were interested in exploring HR-deficient contexts, due to the complementary and compensatory role that HR plays with TLS during replication stress [28]. Moreover, recent reports revealed that HR deficiency is a much more widely spread feature of human cancers than anticipated [23,43], and therefore it is a niche of critical importance for drug discovery and for the design of novel therapeutic strategies.

The rationale we followed was that the sole inhibition of TLS would be insufficient to trigger substantial lethality of HR-deficient cells, and therefore should be combined with replication stress inducers such as UV or cisplatin. When we inhibited PCNA ubiquitylation

by AKT inhibition, RAD18 knockdown or using a knock-in model of non-ubiquitylable PCNA, we observed synthetic lethality (SL) induction in BRCA-deficient cells (Figs 6 and 7), thus indicating that the targeted inhibition of DNA damage tolerance pathways is selectively toxic when cells are deficient in homologous recombination. These results are promising and put forward the use of pharmacological TLS inhibitors as sensitizers of widely used replication poisons such as cisplatin, which are currently the standard of care for HR-deficient cancers [23]. Moreover, these findings also suggest that PCNA ubiquitylation inhibitors would be of therapeutic utility to counteract the resistance to cisplatin, which has been linked in the past to the overexpression of TLS polymerases [44–46]. While some currents efforts to inhibit TLS by targeting TLS polymerases have been reported [11], we believe that inhibiting PCNA ubiquitylation should have a more robust effect on TLS inhibition, since it would have a universal effect on the recruitment of TLS polymerases. This notion is supported by our previous work with the TLS inhibitor p21, which is able to block the recruitment of all TLS polymerases to DNA damage sites, thus impacting on TLS efficiency [12,47]. Taken together, our data put forward a novel model of synthetic lethality induction with great therapeutic potential against HR-deficient cancer cells, where TLS inhibition can act as a strong sensitizer for the specific killing of cells submitted to replication stress. Excitingly, in this context our findings also propose a new therapeutic utility for AKT inhibitors that are currently in clinical trials [48,49], which might be used in combination with replication stress inducers in patient cohorts with known HR deficiencies.

Materials and methods

<u>DNA constructs, shRNA and siRNA</u>

The parental GFP-Polη plasmid was a gift from Dr. Alan Lehmann. GFP-H-Polη was obtained by cloning a flexible hydrophilic linker between Polη and GFP using XhoI restriction site [50]. For stable expression, GFP-H-Polη was cloned into pLenti (w175-1) vector through BamHI and XbaI restriction sites. shRAD18 lentiviral vectors were purchased from Origene (#1: TL302132C; #2: TL302132B; #3: TL302132D; #4: TL302132A). shBRCA1 (TRCN0000010305, Sigma-Aldrich) was cloned into pLKO.1-TRC vector through EcoRI and AgeI restriction sites; and shSCR-pLKO.1 was purchase from Addgene (ID#1864). The siRNA duplexes used (Cell Signaling Technology) were: siSCR (control) 6568S and siAKT 6211S.

<u>Antibodies</u>

Primary antibodies used were: α-ubiquityl-PCNA (D5C7P; Cat# 13439), α-PCNA (PC-10; Cat# 2586), α-pan-Akt (Cat# 4691), α-phospho-Akt (Ser473; Cat# 9271), α-phospho-GSK3B (Ser9; Cat# 9336), α-phospho-PRAS40 (Thr246; Cat# 2997), α-RAD18 (Cat# 9040) and α-SMC-1 (Cat# 4802) from Cell Signaling Technology; α-BRCA1 (Ab-1) from Oncogene Research; α-PCNA (PC-10, Cat# sc-56) from SCBT; α-γH2AX (Cat# 05-636-1) from Millipore; α-CPD (Cat# NMDND001) from Cosmo Bio; α-Tubulin (Cat# T9026) from Sigma-Aldrich. Secondary antibodies used were: α-mouse Alexa Fluor 594 from Jackson ImmunoResearch; goat α-mouse IRDye 680RD (Cat# P/N 925-68070) and goat α-rabbit IRDye 800CW (Cat# P/N 925-32211) from LI-COR Biosciences. Nuclei were stained with DAPI (Cat# D9542) from Sigma-Aldrich.

<u>Cell culture, transfections and UV irradiation.</u>

U2OS, MDA-MB 231 and 436 cell lines were acquired from ATCC. U2OS cells stably expressing DR-GFP were kindly provided by M. Jasin[54]. PCNA wt and PCNA K164R MEF cell lines were previously described by H. Jacobs[25]. HCT116$^{p21-/-}$ were kindly provided by B. Vogelstein. V-C8 cell lines were supplied by B. Lopez. U2OS cell lines were cultured in DMEM medium (Thermo Fisher Scientific) supplemented with 5% FBS (GIBCO). Remaining cell lines were cultured in DMEM medium supplemented with 10% FBS. HEK293T cells were transfected to obtain virus particles using JetPrime (Polyplus-transfection) according to manufacturer’s instructions. siRNAs (100–200 nM) were transfected into cells at 40% confluence, using JetPrime (Polyplus-transfection). Local and global UV irradiation was performed as previously described [51]. All the cell lines used in this work were negative for mycoplasma contamination.

<u>*Protein analysis*</u>

For direct western blot analysis, samples were lysed in commercial Laemmli buffer (BioRad) with reducing agent 2-mercaptoethanol. The detection and quantification were performed with Odyssey Clx System (LI-COR Biosciences) through the Image Studio Software.

<u>*Immunofluorescence and image analysis*</u>

Immunofluorescence and CPD staining were performed as described previously [51]. For GFP-H-Pol η foci detection, cells were pre-extracted with 0.1% Triton for 5 min on ice prior fixation. This method allows detection of only well-assembled foci. Images were captured using an optical microscope equipped with a motorized stage (Leica DMI 8). To quantify the total GFP or γH2AX fluorescence, an Image J Macro was developed using DAPI for nuclei identification and segmentation.

Cell-cycle and cell death analysis.

For cycle analysis, cells were prepared as described previously [52]. SYTOX Red (Thermo Fisher Scientific) was used for dead cell staining according as previously described [53]. Stained samples were subjected to FACS (Attune NxT, Thermo Fisher Scientific) and data were analyzed using FlowJo software (FlowJo LLC). When indicated, the profiles shown were obtained by gating the positive cells by dual-channel FACS analysis.

Preparation and immunolabelling of DNA combing

DNA combing was performed according to our previously described protocol[12] with modifications. Briefly, cells were irradiated with 15 J/m^2 UVC and treated or not with C11 (0.5 μM). After 16 h of treatment, cells were pulse labeled with CldU (20 mM) for 10 min, washed twice, and incubated with IdU (200 mM) for additional 30 min (200 mM). DNA fibers were visualized using a Zeiss Axioplan confocal microscope. Images were analyzed using Zeiss LSM Image Browser software. Only bi-colored fibers were quantified to ensure that only active replication forks, but neither terminations nor recently fired origins, were analyzed.

Clonogenic assay

750 $HCT116^{p21-/-}$ shSCR or shBRCA1 cells were plated in a 96 MW format. Cells were treated with a combination of AKT inhibition (C11 0.1 uM) and UV irradiation and after 6 days the survival fraction was stained with crystal violet.

Homologous recombination analysis

We used an HR assay generated previously in U2OS cells containing an integrated HR reporter substrate DR-GFP [54] with some modifications described previously [52] .

Statistical analysis

All experiments were performed by duplicate or triplicate. Graphs and statistical analysis were performed using GraphPad Prism 5.0 (GraphPad Software), applying two-sided Student's t-test and ANOVA test as appropriate. Bars represent the mean value ± s.d. Other calculations were performed using Microsoft Excel 2003.

Acknowledgments

We thank the flow cytometry, microscopy and cell culture facilities of CIBICI-CONICET for technical support. We thank Nicolas Nieto Moreno for careful reading of the manuscript and helpful suggestions. We would like to thank Gerard Drewes of CellZome (a GSK company) for his scientific advice. This work was supported by a Start-up grant from the Fondo para la Investigación Científica y Tecnológica (FONCyT PICT start-up 2013-1553), by KWF projects 10796/2016-2 and 10032/2016-1 to H. J. and by a consortium grant of FONCyT and the Trust in Science (Global Health R&D) from GlaxoSmithKline (PAE-GLAXO 2014-0005). S.M, V.G., J.L.B. and G.S. are researchers from CONICET. F.V., I.A.G., S.C. and M.C.L were supported by fellowships from FONCyT, M.F.P. and L.G. were supported by fellowships from the National Institute of Cancer and CONICET.

Conflict of interest

No conflict of interest reported

Bibliography

1 Hoege C, Pfander B, Moldovan G-L, Pyrowolakis G, Jentsch S. RAD6-dependent DNA repair is linked to modification of PCNA by ubiquitin and SUMO. *Nature* 2002; **419**: 135–141.

2 Soria G, Podhajcer O, Prives C, Gottifredi V. P21Cip1/WAF1 downregulation is required for efficient PCNA ubiquitination after UV irradiation. *Oncogene* 2006; **25**: 2829–2838.

3 Niimi A, Brown S, Sabbioneda S, Kannouche PL, Scott A, Yasui A *et al.* Regulation of proliferating cell nuclear antigen ubiquitination in mammalian cells. *Proc Natl Acad Sci U S A* 2008; **105**: 16125–16130.

4 Brun J, Chiu RK, Wouters BG, Gray DA. Regulation of PCNA polyubiquitination in human cells. *BMC Res Notes* 2010; **3**. doi:10.1186/1756-0500-3-85.

5 Wit N, Buoninfante OA, Van Den Berk PCM, Jansen JG, Hogenbirk MA, De Wind N *et al.* Roles of PCNA ubiquitination and TLS polymerases κ and η in the bypass of methyl methanesulfonate-induced DNA damage. *Nucleic Acids Res* 2015; **43**: 282–294.

6 Bienko M, Green CM, Crosetto N, Rudolf F, Zapart G, Coull B *et al.* Ubiquitin-binding domains in Y-family polymerases regulate translesion synthesis. *Science* 2005; **310**: 1821–1824.

7 McIntyre J, Woodgate R. Regulation of translesion DNA synthesis: Posttranslational modification of lysine residues in key proteins. *DNA Repair (Amst)* 2015; **29**: 166–179.

8 Soria G, Gottifredi V. PCNA-coupled p21 degradation after DNA damage: The exception that confirms the rule? *DNA Repair (Amst)* 2010; **9**: 358–364.

9 Bertolin AP, Mansilla SF, Gottifredi V. The identification of translesion DNA synthesis regulators: Inhibitors in the spotlight. *DNA Repair (Amst)* 2015; **32**: 158–164.

10 Vaisman A, Woodgate R. Translesion DNA polymerases in eukaryotes: what makes them tick? Crit. Rev. Biochem. Mol. Biol. 2017; **52**: 274–303.

11 Yamanaka K, Chatterjee N, Hemann MT, Walker GC. Inhibition of mutagenic translesion synthesis: A possible strategy for improving chemotherapy? *PLoS Genet* 2017; **13**. doi:10.1371/journal.pgen.1006842.

12 Mansilla SF, Soria G, Vallerga MB, Habif M, Martinez-López W, Prives C *et al.* UV-triggered p21 degradation facilitates damaged-DNA replication and preserves genomic stability. *Nucleic Acids Res* 2013; **41**: 6942–6951.

13 Manning BD, Toker A. AKT/PKB Signaling: Navigating the Network. Cell. 2017; **169**: 381–405.

14 Manning BD, Cantley LC. AKT/PKB signaling: navigating downstream. *Cell* 2007; **129**: 1261–74.

15 Song G, Ouyang G, Bao S. The activation of Akt/PKB signaling pathway and cell survival. *J Cell Mol Med* 2005; **9**: 59–71.

16 Sabbioneda S, Gourdin AM, Green CM, Zotter A, Giglia-Mari G, Houtsmuller A *et al.* Effect of proliferating cell nuclear antigen ubiquitination and chromatin structure on the dynamic properties of the Y-family DNA polymerases. *Mol Biol Cell* 2008; **19**: 5193–202.

17 Wu YT, Ouyang W, Lazorchak AS, Liu D, Shen HM, Su B. mTOR complex 2 targets Akt for proteasomal degradation via phosphorylation at the hydrophobic

motif. *J Biol Chem* 2011; **286**: 14190–14198.

18 Han EKH, Leverson JD, McGonigal T, Shah OJ, Woods KW, Hunter T *et al.* Akt inhibitor A-443654 induces rapid Akt Ser-473 phosphorylation independent of mTORC1 inhibition. *Oncogene* 2007; **26**: 5655–5661.

19 Martini M, De Santis MC, Braccini L, Gulluni F, Hirsch E. PI3K/AKT signaling pathway and cancer: An updated review. Ann. Med. 2014. doi:10.3109/07853890.2014.912836.

20 Tu Y, Ji C, Yang B, Yang Z, Gu H, Lu CC *et al.* DNA-dependent protein kinase catalytic subunit (DNA-PKcs)-SIN1 association mediates ultraviolet B (UVB)-induced Akt Ser-473 phosphorylation and skin cell survival. *Mol Cancer* 2013. doi:10.1186/1476-4598-12-172.

21 Huang TT, Nijman SMB, Mirchandani KD, Galardy PJ, Cohn MA, Haas W *et al.* Regulation of monoubiquitinated PCNA by DUB autocleavage. *Nat Cell Biol* 2006. doi:10.1038/ncb1378.

22 Sale JE, Lehmann AR, Woodgate R. Y-family DNA polymerases and their role in tolerance of cellular DNA damage. *Nat Rev Mol Cell Biol* 2012; **13**: 141–152.

23 Lord CJ, Ashworth A. BRCAness revisited. *Nat Rev Cancer* 2016; **16**: 110–120.

24 Zhang W, Qin Z, Zhang X, Xiao W. Roles of sequential ubiquitination of PCNA in DNA-damage tolerance. FEBS Lett. 2011; **585**: 2786–2794.

25 Langerak P, Nygren AOH, Krijger PHL, van den Berk PCM, Jacobs H. A/T mutagenesis in hypermutated immunoglobulin genes strongly depends on PCNA ^{K164} modification. *J Exp Med* 2007; **204**: 1989–1998.

26 Sokol AM, Cruet-Hennequart S, Pasero P, Carty MP. DNA polymerase η modulates replication fork progression and DNA damage responses in platinum-treated human cells. *Sci Rep* 2013; **3**. doi:10.1038/srep03277.

27 Jackson SP, Bartek J. The DNA-damage response in human biology and disease. *Nature* 2009; **461**: 1071–1078.

28 Sale JE. Competition, collaboration and coordination--determining how cells bypass DNA damage. *J Cell Sci* 2012; **125**: 1633–1643.

29 Branzei D, Szakal B. DNA damage tolerance by recombination: Molecular pathways and DNA structures. DNA Repair (Amst). 2016; **44**: 68–75.

30 Cipolla L, Maffia A, Bertoletti F, Sabbioneda S. The regulation of DNA damage tolerance by ubiquitin and ubiquitin-like modifiers. Front. Genet. 2016; **7**. doi:10.3389/fgene.2016.00105.

31 Surucu B, Bozulic L, Hynx D, Parcellier A, Hemmings BA. In vivo analysis of protein kinase B (PKB)/Akt regulation in DNA-PKcs-null mice reveals a role for PKB/Akt in DNA damage response and tumorigenesis. *J Biol Chem* 2008; **283**: 30025–30033.

32 Park J, Feng J, Li Y, Hammarsten O, Brazil DP, Hemmings BA. DNA-dependent protein kinase-mediated phosphorylation of protein kinase B requires a specific recognition sequence in the C-terminal hydrophobic motif. *J Biol Chem* 2009; **284**: 6169–6174.

33 Boehme KA, Kulikov R, Blattner C. p53 stabilization in response to DNA damage requires Akt/PKB and DNA-PK. *Proc Natl Acad Sci* 2008; **105**: 7785–7790.

34 Wang Z, Huang M, Ma X, Li H, Tang T, Guo C. REV1 promotes PCNA monoubiquitylation through interacting with ubiquitylated RAD18. *J Cell Sci*

2016; **129**: 1223–1233.

35 Madia F, Wei M, Yuan V, Hu J, Gattazzo C, Pham P *et al.* Oncogene homologue Sch9 promotes age-dependent mutations by a superoxide and Rev1/Polζ-dependent mechanism. *J Cell Biol* 2009; **186**: 509–523.

36 Jia J, Zhang Y, Cai J, Wang J, Ding H, Zhou J *et al.* A novel function of protein kinase B as an inducer of the mismatch repair gene hPMS2 degradation. *Cell Signal* 2013; **25**: 1498–1504.

37 Liu Q, Turner KM, Yung WKA, Chen K, Zhang W. Role of AKT signaling in DNA repair and clinical response to cancer therapy. *Neuro Oncol* 2014; **16**: 1313–1323.

38 Despras E, Daboussi F, Hyrien O, Marheineke K, Kannouche PL. ATR/Chk1 pathway is essential for resumption of DNA synthesis and cell survival in UV-irradiated XP variant cells. *Hum Mol Genet* 2010; **19**: 1690–1701.

39 Despras E, Delrieu N, Garandeau C, Ahmed-Seghir S, Kannouche PL. Regulation of the specialized DNA polymerase eta: Revisiting the biological relevance of its PCNA- and ubiquitin-binding motifs. *Environ Mol Mutagen* 2012; **53**: 752–765.

40 Hendel A, Krijger PHL, Diamant N, Goren Z, Langerak P, Kim J *et al.* PCNA ubiquitination is important, but not essential for translesion DNA synthesis in mammalian cells. *PLoS Genet* 2011; **7**: e1002262.

41 Moldovan G-LL, Pfander B, Jentsch S. PCNA, the Maestro of the Replication Fork. *Cell* 2007; **129**: 665–679.

42 Wang SC. PCNA: A silent housekeeper or a potential therapeutic target? Trends Pharmacol. Sci. 2014; **35**: 178–186.

43 Davies H, Glodzik D, Morganella S, Yates LR, Staaf J, Zou X *et al.* HRDetect is a predictor of BRCA1 and BRCA2 deficiency based on mutational signatures. *Nat Med* 2017; **23**: 517–525.

44 Ceppi P, Novello S, Cambieri A, Longo M, Monica V, Lo Iacono M *et al.* Polymerase eta mRNA Expression Predicts Survival of Non Small Cell Lung Cancer Patients Treated with Platinum-Based Chemotherapy. *Clin Cancer Res* 2009; **15**: 1039–1045.

45 Sharma S, Shah N a, Joiner a M, Roberts KH, Canman CE. DNA polymerase zeta is a major determinant of resistance to platinum-based chemotherapeutic agents. *Mol Pharmacol* 2012; **81**: 778–787.

46 Srivastava AK, Han C, Zhao R, Cui T, Dai Y, Mao C *et al.* Enhanced expression of DNA polymerase eta contributes to cisplatin resistance of ovarian cancer stem cells. *Proc Natl Acad Sci U S A* 2015; **112**: 4411–4416.

47 Soria G, Speroni J, Podhajcer OL, Prives C, Gottifredi V. p21 differentially regulates DNA replication and DNA-repair-associated processes after UV irradiation. *J Cell Sci* 2008; **121**: 3271–3282.

48 Nitulescu GM, Margina D, Juzenas P, Peng Q, Olaru OT, Saloustros E *et al.* Akt inhibitors in cancer treatment: The long journey from drug discovery to clinical use (Review). Int. J. Oncol. 2016; **48**: 869–885.

49 Brown JS, Banerji U. Maximising the potential of AKT inhibitors as anti-cancer treatments. Pharmacol. Ther. 2017; **172**: 101–115.

50 Leonhardt H, Rahn HP, Weinzierl P, Sporbert A, Cremer T, Zink D *et al.* Dynamics of DNA replication factories in living cells. *J Cell Biol* 2000; **149**: 271–80.

51 Soria G, Belluscio L, van Cappellen WA, Kanaar R, Essers J, Gottifredi V. DNA damage induced Pol eta recruitment

takes place independently of the cell cycle phase. *Cell Cycle* 2009; **8**: 3340–3348.

52 Soria G, Almouzni G. Differential contribution of HP1 proteins to DNA end resection and homology-directed repair. *Cell Cycle* 2013; **12**: 422–429.

53 Joray M, Villafanez F, Gonzales M, Crespo M, Laiolo J, Palacios S *et al.* P53 tumor suppressor is required for efficient execution of the death program following treatment with a cytotoxic limonoid obtained from Melia azedarach. *Food Chem Toxicol* 2017; : pii: S0278-6915(17)30217-X.

54 Pierce AJ. Ku DNA end-binding protein modulates homologous repair of double-strand breaks in mammalian cells. *Genes Dev* 2001; **15**: 3237–3242.

Figure legends

Figure 1: Miniaturized western blot setup to perform a screening of PCNA ubiquitylation inhibitors. A) U2OS cells were UV-irradiated (15 J/m2) and treated for 12 h with the proteasome inhibitors Epoxomicin and MG-132. The western blot was performed with two monoclonal antibodies to simultaneously detect total PCNA (in red) and ubi-PCNA (in green) using a LICOR Odyssey infrared scanner. The ratios of ubi-PCNA/total PCNA were normalized to the highest induction of ubi-PCNA in the non-treated (NT) UV-irradiated sample. **B)** 3 days detailed protocol to screen for PCNA ubiquitylation inhibitors, showing the quality controls to ensure reproducibility and robustness of PCNA ubiquitylation induction: i) use of an infrared scanner to confirm the homogenous distribution of cells in the wells across the entire plate before the addition of the screening compounds ; ii) Automatized capture of a low magnification brightfield image at the center of each well as a control of the general cytotoxicity of every treatment ; iii) Lysis in benzonase w/o boiling of the samples and direct loading of the samples to the SDS Page gel**C)** Layout of the 96 multi-well (MW) plates used in the screening, showing the disposition of the non-irradiated and UV-irradiated controls. 80 Kinase inhibitors per plate were evaluated and 8 mini-western blots were run in parallel with the 12 samples from each plate row. **D)** Results of the screening with 627 kinase inhibitors from the PKIS2 library, tested at 1 µM. The distribution of the normalized ubi-PCNA/total PCNA ratios is shown. The dotted line represents the threshold of 3 standard deviations that allowed the identification of 22 hits.

Figure 2: AKT inhibition impairs PCNA ubiquitylation. A) U2OS cells were UV-irradiated (15J/m2) and treated for 12 h with the indicated inhibitors at 1 µM. The western blot shows the strong

PCNA ubiquitylation inhibitory activity found in two structurally related hits: C11 (compound #: GSK1581428A) and G8 (compound #: GSK1389063A). The graph in the lower panel shows the quantification of three independent experiments. Statistical analysis was performed using ANOVA with Tukey Kramer post-test (***: $p \leq 0.001$). **B)** U2OS cells were treated as in A) and western blots with specific antibodies were performed to study pAKT, total AKT and p-GSK3β levels. α-tubulin was used as a loading control. **C)** U2OS cells were pre-treated for 12 hours using 0.5 uM C11 and 5 uM of the structurally unrelated AKT inhibitors: MK-2206 (Merck), AZD5363 (AstraZeneca), GSK690693 (GlaxoSmithKline). After UV, all these inhibitors were used at 20 uM and C11 was used at 1 uM. The normalized ubi-PCNA/total PCNA ratios are shown below the PCNA panel. pAKT, AKT, p-GSK3β and p-PRAS40 western blots were performed at 3 and 12 h post-treatment to confirm the AKT inhibitory activity of each compound. **D)** U2OS cell were transfected with two concentrations of siRNAs. 48 h later cells were UV-irradiated, and after 12h samples were processed for quantification of PCNA ubiquitylation by western blot. A western blot for pan-AKT was performed to confirm the siRNA mediated knockdown. The normalized ubi-PCNA/total PCNA ratios are shown below the PCNA panel.

Figure 3: AKT inhibition impairs PCNA ubiquitylation only in the context of replication stress.

A) U2OS cells were pre-treated overnight with the indicated kinase inhibitors: PI3K inhibitor (LY294002 50 uM), ATR inhibitor (VE-821 1 uM), ATM inhibitor (KU-55933 1 uM), DNA PKcs inhibitor (NU7026 20 uM) and AKT inhibitor (C11 1 uM). Cells were then submitted to UV irradiation (15 J/m2), and 12 h in the presence of the inhibitors at the same concentrations, samples were processed by WB for the quantification of ubi/PCNA. **B)** U2OS cells were treated in

parallel and in combination of suboptimal doses of the AKT inhibitor C11 (0.1 uM) and the optimal dose of the DNA PKcs inhibitor NU7026 (20 uM). 12 hs after UV irradiation (15 J/m2) samples were processed by WB for the quantification of ubi/PCNA. **C)** U2OS cells were treated overnight with the optimal dose of each kinase inhibitor (LY294002 50 uM, NU7026 20 uM and C11 1 uM) followed by sample processing for WB in unperturbed conditions. **D)** U2OS cells were transfected with 75 nM of siRNA against USP1. 48 h later cells were treated with C11 1 uM and after 12h samples were processed for quantification of PCNA ubiquitylation by western blot.

Figure 4: AKT inhibition impair TLS activation markers and replication fork processivity. A) Schematic representation of the local UV irradiation method using 5 µm micropore filters as UV shields. **B)** U2OS cells stably transduced with GFP-H-Pol η were pre-treated with the AKT inhibitor C11 (0.5 µM) for 10 h and locally UV-irradiated (100 J/m2) in discrete areas of the nuclei as indicated in A). Immediately after irradiation cells were re-incubated with the AKT inhibitor for 4 h and fixed. IFs were performed to detect cyclobutane pyrimidine dimers (CPDs), which delimitate the damaged DNA areas. The quantification was performed by counting the CPDs positive cells that show focal accumulation of GFP-H-Pol η. The results of three independent experiments are shown, in which at least 200 cells/condition were analyzed. **C)** Schematic representation of the global UV irradiation method. **D)** U2OS cells stably transduced with GFP-H-Pol η were UV irradiated (40 J/m2) and incubated with C11 (0.5 µM). 4 h later, immediately prior to fixation, cells were treated with PBS 0.1% Triton to wash out the soluble fraction of GFP-H-Pol η. An Image J macro was developed to unbiasedly quantify the remaining GFP-H-Pol η fraction in each experimental condition. DAPI staining was used to segment the nuclei and at least 1000

cells/condition were analyzed. The right panel shows the average of three independent experiments. **E)** Detailed DNA combing protocol used to evaluate the effect of AKT inhibition on the processivity of replication forks submitted to UV irradiation. Only bi-color fibers were measured to ensure that only active replication replication forks were analyzed **F)** DNA bi color fibers were imaged in each condition using confocal microscopy and were manually measured using Image J. The total length of 200 DNA fibers/condition are shown on the right panel. Statistical analysis shown in Figure B, D and F were performed using the T- test (**: $p \leq 0.01$; ***: $p \leq 0.001$).

Figure 5: AKT inhibition triggers replication stress-associated DNA damage and cell death after UV irradiation. A) U2OS cells were UV irradiated (15 J/m2) and 24 h later cells were fixed and γH2AX immunostaining was performed. An Image J macro was developed to quantify only the nuclear γH2AX signal using DAPI for segmentation. Two doses of the AKT inhibitor C11 were used. The right panel show the average of two independent experiments were at least 100 cells /condition were analyzed. Statistical analysis was performed using ANOVA with Tukey Kramer post- test (*: $p \leq 0.05$). **B)** U2OS cells were UV-irradiated (15 J/m2) and treated with C11 (0.5 uM). 24 h later cells were processed for cell cycle analysis by flow cytometry using propidium iodide (PI). Cell cycle analysis was performed using Flowjo Tree Star, Inc. software. The right panel show the determination of the relative % of each cell cycle phase, including the apoptotic sub-G1 population. **C)** U2OS cells were UV irradiated (15 J/m2) and treated with the AKT inhibitor C11 (0.5 uM). 24 h later cells were stained using the dead cells stain Sytox red and cells were

immediately processed by flow cytometry. The % of live and dead cells was determined for each condition.

Figure 6: AKT inhibition is synthetic lethal with the homologous recombination deficiency induced by BRCA1 knockdown. A) Experimental layout and detailed protocol used to assess synthetic lethality (SL) induction using a co-culture method of HR+ and HR- isogenic HCT116$^{p21-/-}$ cell lines, generated by lentiviral transduction of shRNAs against BRCA1. Each cell line co-expresses a different fluorescent protein: shSCR (CFP) and shBRCA1 (iRFP). Equal numbers of both isogenic cells were then plated in triplicates in 96 MW plates and combinations of increasing UV doses with AKT inhibitors were performed. 6 days post treatment the co-cultured population was counted and categorized by the differential expression of fluorescent proteins using automated flow cytometry with an autosampler. The remaining % of each cell population was determined and the ratio of HR-/HR+ cells was calculated. The relative survival of each cell population in comparison to the untreated controls was determined to calculate SL induction by the different treatments. **B)** Positive control to calibrate the robustness of the SL induction assay at 6 days using the PARP inhibitor Olaparib (0.1 µM), which is selectively toxic against the HR- population. **C)** Determination of SL induction using HR+ (shSCR) and HR- (shBRCA1) isogenic HCT116$^{p21-/-}$ cells in a dose-response UV irradiation curve combined with three AKT inhibitors after 6 days of treatment: C11 (0.1 uM), MK-2206 (1 uM) and AZD5363 (1 uM). Statistical analysis shown in Figure B and C was performed using ANOVA (*: $p \leq 0.05$; **: $p \leq 0.01$; ***: $p \leq 0.001$). **D)** Clonogenic experiments comparing HCT116$^{p21-/-}$ shSCR vs. shBRCA1 cells treated with the combination of AKT inhibition (C11 0.1 uM) and UV irradiation. Duplicates of each treatment are

displayed. 750 cells were plated in a 96 MW format and after 6 days the survival fraction was stained with crystal violet. **E)** Control WBs confirming the efficient impairment of PCNA ubiquitylation after UV triggered by AKT inhibition for every cell line used to validate the induction of SL. In the case of mouse and hamster cells (MEF and V-C8), the detection of PCNA ubiquitylation was performed using the total PCNA antibody (PC-10) because the Ubiquityl-PCNA antibody (D5C7P) only reacts with human samples. **F)** Determination of SL induction using HR+ and HR- cells (BRCA1 or BRCA2 deficient) at the optimal UV irradiation dose in combination with the AKT inhibitor C11 (1 uM). A pair of triple negative breast cancer cell lines (MDA-MB 231-BRCA1wt vs MDA-MB 436-BRCA1KO), a set of wt MEF (shSCR vs shBRCA1), a hamster BRCA2 KO cell line with its reconstituted counterpart (VC8 vs VC#13) and a pair of HCT116$^{p21-/-}$ cells (shSCR vs shBRCA2) were used. Statistical analysis shown in panels B, C and F was performed using ANOVA (*: $p \leq 0.05$; **: $p \leq 0.01$; ***: $p \leq 0.001$).

Figure 7: Abrogation of PCNA ubiquitylation triggers synthetic lethality in HR-deficient cells submitted to replication stress. A) HCT116$^{p21-/-}$ cells were transduced with 4 different shRNAs against RAD18 at increasing multiplicities of infection (MOI). 72 h later cells were processed for western blot to detect RAD18. **B)** HCT116$^{p21-/-}$ cells were transduced with the most efficient RAD18 shRNAS (#1 and #4). After 72 h, cells were UV irradiated (15 J/m2) and the induction of PCNA ubiquitylation was analyzed by western blot 12 h later. **C)** Detailed protocol used to assess SL induction after RAD18 knockdown. Cells were plated in 6MW plates and transduced with lentiviral shRNAs #1 or #4. 48 h later cells were re-plated into a 96 MW format. 24 h later cells were UV irradiated. 6 days post UV irradiation the determination of SL induction using HR+ and

HR- cells was performed by calculating the relative survival of the cells transduced with the shRNA against RAD18 in comparison to the non-transduced cells. Such differential analysis was possible by gating the transduced population due to the concomitant expression of GFP with the shRNAs. **D)** HR+ and HR- cells were transduced with shRNA #1 using the protocol detailed in C. The relative survival of the transduced population was calculated using the non-irradiated population as control. A dose response UV curve was performed, and samples were processed using 8 experimental replicates. **E)** PCNA wt and PCNA K164R mouse embryonic fibroblasts (MEFs) were UV-irradiated (40 J/m2). 12 h after UV irradiation samples were processed for WB and ubi-PCNA induction was assessed using a monoclonal antibody that detects mouse PCNA. **F)** PCNA wt and PCNA K164R MEFs were transduced with shRNAs against murine BRCA1. Each set of cells were UV-irradiated following a dose response curve with or without treatment with the AKT inhibitor C11. After 6 days the relative survival of each cell population was determined using automated flow cytometry. **G)** HR+ and HR- cell pairs were treated with cisplatin and after 1 h the culture media was replaced. The relative survival of the different HR- vs HR+ pairs was assessed 6 days later **H)** PCNA wt and PCNA K164R MEFs were treated with cisplatin and after 1 h the culture media was replaced. The relative survival of the different HR- vs HR+ pairs was assessed 6 days later. Statistical analysis shown in panels D, F, G and H was performed using ANOVA (*: $p \leq 0.05$; **: $p \leq 0.01$; ***: $p \leq 0.001$).

Supplementary Figure 1: Single antibody vs dual antibody detection of total and ubi-PCNA.

A) U2OS cells were grown in a 24 MW format. 100K cells were seeded per 24 well. 24 h later cells were UV-irradiated (40 J/m2) and 4 or 8 h post UV cells were processed for WB. Sample lysis was

performed with Laemmli-buffer, collected in 1.5 ml tubes and boiled for 15 minutes. The total sample volume for each treatment was loaded in a WB and PCNA and ubi-PCNA detection was performed using a monoclonal antibody against PCNA (clone PC10). **B)** U2OS cells were grown in a 96 MW format. 30K cells were seeded per 96 well. 24 h later samples were UV irradiated (40 J/m2) and 4 h post UV samples were processed for WB. Sample lysis was performed in the same MW plate using benzonase (250U/mL). Loading of WB gels was performed directly from the 96 MW plate without boiling. The total sample volume from each well was loaded and the detection of PCNA and ubi-PCNA was performed by the simultaneous incubation with two monoclonal antibodies from cell signaling (mouse α PCNA, clone PC10, and rabbit α ubi-PCNA clone D5C7P).

Supplementary Figure 2: Identification and validation of the AKT inhibitors GSK1581428A and GSK1389063A. A) Screening results of the 96 MW plate containing compounds GSK1581428A and GSK1389063A, which were named C11 and G8 respectively, due to their positions in the plate. The quantification was performed using the software of the LICOR Odyssey infrared scanner and the inhibitory capacity of the compounds was calculated by comparing to the normalized values of the 8 non-treated (UV-irradiated) of the plate. **B)** U2OS cells were UV irradiated (15 J/m2) and treated with increasing doses of compound C11, from 0.1 µM to 1 µM. 12 h later, the inhibition of PCNA ubiquitylation and AKT activation were analyzed by western blot.

Supplementary Figure 3: Tools to validate TLS impairment after AKT inhibition. A) U2OS cells were either transiently transfected or stably transduced with GFP-H- Pol η using lentiviral vectors.

A low magnification image (20 X) using a fluorescence microscope was taken to show the homogeneity and levels of GFP-H- Pol η in each case. The transient expression was evaluated after 48 h and the stable expression was evaluated two weeks after lentiviral transduction and puromycin selection. **B)** DAPI images corresponding to Figure 4A, which were used to segment the nuclei prior to γH2AX quantification.

Supplementary Figure 4: Induced HR deficiencies in the isogenic cell lines generated to evaluate the impact of impaired PCNA ubiquitylation. A) Western blot showing the efficiency of BRCA1 knockdown using a lentiviral shRNA approach. The pair of isogenic HCT116$^{p21-/-}$ cells obtained (shSCR and shBRCA1) were used in all the experiments shown in Figures 6 and 7. **B)** HR deficiency triggered by the same shRNA used in A) tested with the DR-GFP method. The right panel shows the normalized quantification of HR efficiency in shSCR vs shBRCA1 cells. **C)** SL induction experiments showing the complete UV dose response curve of the experiment shown in Figure 6F. **D)** Results of the survival experiment using the RAD18 shRNA #4, performed in parallel with shRNA #1 (shown in Figure 7D). Statistical analysis shown in panels C and D was performed using ANOVA (*: $p \leq 0.05$; **: $p \leq 0.01$; ***: $p \leq 0.001$).
